# *Babesia* spp. in questing ticks from eastern Poland: prevalence and species diversity

**DOI:** 10.1007/s00436-015-4529-5

**Published:** 2015-05-16

**Authors:** Angelina Wójcik-Fatla, Violetta Zając, Anna Sawczyn, Ewa Cisak, Jacek Dutkiewicz

**Affiliations:** Department of Zoonoses, Institute of Rural Health, Jaczewskiego 2, 20-090 Lublin, Poland

**Keywords:** *Babesia* spp., Ticks, *Ixodes ricinus*, *Dermacentor reticulatus*

## Abstract

A total of 853 questing *Ixodes ricinus* males, females, and nymphs and of 582 questing *Dermacentor reticulatus* males and females were collected from vegetation on the territory of the Lublin province (eastern Poland). The ticks were examined for the presence of *Babesia* by PCR detecting part of 18S ribosomal RNA (rRNA) gene and nuclear small subunit rRNA (SS-rDNA) for determining of *Babesia* spp. and *Babesia microti*, respectively. The overall incidence of *Babesia* strains in *I. ricinus* ticks was 4.6 %. Three species of *Babesia* were identified. The prevalent species was *B. microti* which occurred in 2.8 % of ticks, while *Babesia venatorum*, *Babesia divergens*, and unidentified *Babesia* species were found at the frequency of 1.2, 0.2, and 0.3 %, respectively. Altogether, *B. microti* constituted 61.5 % of the total strains detected in *I. ricinus*, *B. venatorum*—25.7 %, *B. divergens*—5.1 %, and unidentified *Babesia* species—7.7 %. The prevalence of *Babesia* species in *I. ricinus* did not depend significantly on locality (*χ*^2^ = 1.885, *P* = 0.390) nor on the tick stage (*χ*^2^ = 4.874, *P* = 0.087). The incidence of *Babesia* strains in *D. reticulatus* ticks was 2.7 %. Two species of *Babesia* were identified. Again, the prevalent species was *B. microti* which occurred in 2.1 % of ticks, while *B. canis* was found in 0.7 % of ticks. In one *D. reticulatus* female, *B. canis* and *B. microti* co-infection was found. Altogether, *B. microti* constituted 75 % of the total strains detected in *D. reticulatus* while *B. canis* formed 25 % of the total strains. The frequency of the occurrence of *Babesia* species in *D. reticulatus* did not depend significantly on locality (*χ*^2^ = 0.463, *P* = 0.793). The difference between the prevalence of *Babesia* in males and females of *D. reticulatus* was insignificant (*P* = 0.0954); nymphs were not found. The dominance of *B. microti* in the species composition of tick-borne *Babesia* found in this study was typical for eastern Europe. In conclusion, the results revealed that the population inhabiting the forested area of eastern Poland could be exposed to *Babesia* parasites, especially to those from the species *B. microti*, by a bite of *I. ricinus*, a competent vector of human babesiosis, and probably also by a bite of *D. reticulatus* whose role in the transmission of human babesiosis needs to be clarified.

## Introduction

The protozoan genus *Babesia* Starcovici, 1893 (Apicomplexa: Piroplasmida: Babesiidae) comprises intraerythrocytic parasites of mammals and birds which are transmitted by hard ticks (Ixodidae) from the genera *Amblyomma, Boophilus*, *Dermacentor*, *Haemaphysalis*, *Hyalomma*, *Ixodes*, and *Rhipicephalus*. These hemoprotozoans cause babesiosis, a disease of animals and humans manifested in severe cases by fever and hemolysis leading to anemia, hyperbilirubinuria, hemoglobinuria, and possible organ failure (Peirce 2000; Hunfeld and Brade 2004; Hamel et al. 2012; Altay et al. 2012; Hildebrandt and Hunfeld 2014; Aydin et al. 2015). To date, more than 100 *Babesia* species have been identified worldwide, of which the most important parasites of domestic animals are *Babesia bigemina*, *Babesia bovis*, and *Babesia divergens* in cattle and *Babesia canis* (formerly *Babesia canis canis*) in dogs. Babesiosis in humans is regarded as an emerging disease with the greatest number of cases (above 1000 per annum) caused by *Babesia microti* in North America (Yabsley and Shock 2012). The disease, transmitted by *Ixodes scapularis*, can range from asymptomatic and mild infections to severe disease and death. In Europe, about 50 cases of babesiosis have been recorded up to date, caused primarily by *B. divergens* in splenectomized individuals, less so by *Babesia venatorum* (formerly *Babesia* sp. EU1) and *B. microti*. The disease may also develop in the immunocompetent individuals (Martinot et al. 2011).

*Ixodes ricinus* is regarded as the most important vector of *Babesia* in Europe; other potential vectors are *Dermacentor reticulatus* and *Ixodes persulcatus* (Hildebrandt et al. 2013; Wójcik-Fatla et al. 2012; Katargina et al. 2011). The aim of the present study was to determine the prevalence and species diversity of *Babesia* in *I. ricinus* and *D. reticulatus* ticks collected in eastern Poland.

## Materials and methods

### Collection of ticks

A total of 582 questing *D. reticulatus* ticks (341 females and 241 males) and a total of 853 questing *I. ricinus* ticks (314 females, 268 males, and 271 nymphs) were collected during spring/summer season in the years 2011–2012 on the areas of six localities situated in the Lublin province (eastern Poland). *D. reticulatus* ticks were collected on the territory of three localities: Ostrów Lubelski (51° 46′ N, 22° 84′ E), Suchawa (51° 49′ N, 23° 40′ E), and Parczew (51° 64′ N, 22° 90′ E). *I. ricinus* ticks were collected on the territory of Wilków (51° 25′ N, 21° 88′ E), Suchawa, and Dąbrowa (51° 17′ N 22° 57′ E). Ticks were collected by dragging a woolen flag over the lower vegetation and litter along the paths and edges of deciduous and mixed forests, including suburban localities and recreational areas.

### DNA isolation from ticks

Total DNA was isolated from the adult ticks separately and from nymphs in pools of five specimens (Rijpkema et al. 1996) by boiling in 0.7 M ammonium hydroxide and stored at −20 °C for further analysis. Prevalence of infection in nymphs was expressed as the minimum infection rate (MIR) of pools calculated according to Kahl et al. (1989). The concentration of DNA in the isolates was determined with the NanoDrop ND1000 Spectrophotometer (USA). The determined DNA concentrations ranged from 500 to 660 ng/μl for males and from 670 to 880 ng/μl for females of *D. reticulatus* and from 300 to 500 ng/μl for females, from 180 to 330 ng/μl for males, and from 20 to 80 ng/μl for nymphs of *I. ricinus*.

### Detection of *B. microti* DNA by PCR and nested PCR

All tick lysates were examined for the presence of *B. microti* DNA using amplification by PCR and confirmatory re-amplification by nested PCR with the method described previously (Persing et al. 1992) with some modification (Wójcik-Fatla et al. 2012). The primers used in this study are specific for a gene encoding the nuclear small subunit ribosomal RNA (SS-rDNA). As a positive control, DNA extracted from the antigen of *B. microti* from the slide used for detection of antibodies (Fuller Laboratories, Germany) was used, while nuclease-free water was used as a negative control. The amplifications were carried out in a C1000 Thermal Cycler (BioRad, USA).

### Detection of *Babesia* spp., *B. divergens*, and *B. venatorum*

Primers for detection of *Babesia* spp. including bovine *Babesia*: *B. divergens*, *B. bigemina*, *B. major*; *B. venatorum*; *B. canis*; *B. odocoilei*; *B. ovata*; *B. motasi*, and *B. crassa*—and primers for identification of *B. divergens* and *B. venatorum* were described previously by Hilpertshauser et al. (2006).

Each PCR reaction was carried out in a 25-μl reaction volume which contained the following mix of reagents: 0.625 U *Taq* DNA polymerase (Qiagen, USA), 1 × PCR buffer containing 15 mM MgCl_2_, 2.5 μl 2 mM dNTP (final concentration 0.1 mM) (Thermo Scientific, Lithuania), 1.25 μl 10 μM each of primer (Eurogentec, Seraing, Belgium), 2 μl of matrix DNA, and nuclease-free water (Applied Biosystems, USA). Tick lysates confirmed as positive for *B. divergens* and *B. venatorum* were used as positive and nuclease-free water as negative controls. The amplification was carried out in C1000 Thermal Cycler (BioRad, USA) under the following conditions: preincubation at 95 °C for 3 min, 45 cycles, each of 30 s at 94 °C (denaturation), 30 s at 61 °C (primers annealing), and 45 s at 72 °C (elongation). Final elongation was performed for 10 min at 72 °C. Products of amplification were identified in 2 % agarose gel (Prona, Basica LE), after electrophoresis in standard conditions and staining with ethidium bromide solution (2 μg/ml).

### DNA sequencing

DNA sequencing of all *Babesia* spp. positive samples was performed with ABI PRISM 310 Genetic Analyzer (Applied Biosystems, Inc., Foster City, CA, USA) using ABI PRISM Big Dye Terminator v. 3.1. Cycle Sequencing Kits and Big Dye XTerminator Purification Kit (Applied Biosystems). For sequencing of *B. microti* positive samples, the tenfold dilution of amplified DNA was used, and fivefold for *Babesia* spp. The results were compared with sequences in GenBank database using the BLAST software at the National Center for Biotechnology Information (Bethesda, Maryland, USA).

### Statistical analysis

The obtained results were analyzed by *χ*^2^ test and Student’s *t* test, using the STATISTICA v. 6.0 package (Statsoft, Tulsa, OK, USA). The value *p* < 0.05 was considered significant.

## Results

The overall incidence of *Babesia* strains in *I. ricinus* ticks collected in eastern Poland was 4.6 % (Table 1). Three species of *Babesia* were identified. The prevalent species was *B. microti* which occurred in 2.8 % of ticks, while *B. venatorum*, *B. divergens*, and unidentified *Babesia* species were found at the frequency of 1.2, 0.2, and 0.3 %, respectively. Altogether, *B. microti* constituted 61.5 % of the total strains detected in *I. ricinus*, *B. venatorum*—25.7 %, *B. divergens*—5.1 %, and unidentified *Babesia* species—7.7 %. The prevalence of *Babesia* species in *I. ricinus* did not depend significantly on locality (*χ*^2^ = 1.885, *P* = 0.390) nor on the tick stage (*χ*^2^ = 4.874, *P* = 0.087).

**Table 1:** Prevalence of various *Babesia species* in *Ixodes ricinus* ticks collected at three localities in the Lublin province (eastern Poland)

Locality *Babesia* species	Ticks infected/examined (percent)															
	Dąbrowa				Suchawa				Wilków				Total			
	F	M	N^a^	T	F	M	N	T	F	M	N	T	F	M	N	T
*Babesia divergens*	0/179	1/139	0/135	1/453	0/48	0/36		0/84	0/87	0/93	1/136	1/316	0/314	1/268	1/271	2/853
	(0)	(0.7)	(0)	(0.2)	(0)	(0)	N. f.	(0)	(0)	(0)	(0.7)	(0.3)	(0)	(0.4)	(0.4)	(0.2)
*Babesia microti*	9/179	2/139	5/135	16/453	3/48	0/36		3/84	1/87	1/93	3/136	5/316	13/314	3/268	8/271	24/853
	(5.0)	(1.4)	(3.7)	(3.5)	(6.2)	(0)	N. f.	(3.6)	(1.1)	(1.1)	(2.2)	(1.6)	(4.1)	(1.1)	(2.9)	(2.8)
*Babesia venatorum*	5/179	1/139	2/135	8/453	0/48	0/36		0/84	1/87	1/93	0/136	2/316	6/314	2/268	2/271	10/853
	(2.8)	(0.7)	(1.5)	(1.8)	(0)	(0)	N. f.	(0)	(1.1)	(1.1)	(0)	(0.6)	(1.9)	(0.7)	(0.7)	(1.2)
Unidentified *Babesia* species	0/179	0/139	0/135	0/453	0/48	0/36		0/84	0/87	0/93	3/136	3/316	0/314	0/268	3/271	3/853
	(0)	(0)	(0)	(0)	(0)	(0)	N. f.	(0)	(0)	(0)	(2.2)	(0.9)	(0)	(0)	(1.1)	(0.3)
Total	14/179	4/139	7/135	25/453	3/48	0/36		3/84	2/87	2/93	7/136	11/316	19/314	6/268	14/271	39/853
	(7.8)	(2.9)	(5.2)	(5.5)	(6.2)	(0)	N. f.	(3.6)	(2.3)	(2.1)	(5.1)	(3.5)	(6.1)	(2.2)	(5.2)	(4.6)

*F* females, *M* males, *N* nymphs, *T* total, *N. f.* not found

^a^Minimum infection rate calculated according to Kahl et al. (1989)

The incidence of *Babesia* strains in *D. reticulatus* ticks collected in eastern Poland was 2.7 % (Table 2). Two species of *Babesia* were identified. The prevalent species was also *B. microti* which occurred in 2.1 % of ticks, while *B. canis* was found in 0.7 % of ticks. Altogether, *B. microti* constituted 75 % of the total strains detected in *D. reticulatus*, while *B. canis* formed 25 % of the total strains. There was one *D. reticulatus* female co-infected with *B. canis* and *B. microti*. The frequency of the occurrence of *Babesia* species in *D. reticulatus* did not depend significantly on locality (*χ*^2^ = 0.463, *P* = 0.793). The difference between the prevalence of *Babesia* in males and females of *D. reticulatus* was insignificant (*P* = 0.0954); nymphs were not found. However, the difference between the diversity of *Babesia* species among *I. ricinus* and *D. reticulatus* ticks proved to be significant (*P* < 0.05).

**Table 2:** Prevalence of various *Babesia* species in *Dermacentor reticulatus* ticks collected at three localities in the Lublin province (eastern Poland)

Locality *Babesia* species	Ticks infected/examined (percent)															
	Ostrów Lubelski				Parczew				Suchawa				Total			
	F	M	N	T	F	M	N	T	F	M	N	T	F	M	N	T
*Babesia canis*	1/81	0/67		1/148	0/147	0/135		0/282	2/113	1/39		3/152	3/341	1/241		4/582
	(1.2)	(0)	N. f.	(0.7)	(0)	(0)	N. f.	(0)	(1.8)	(2.6)	N. f.	(2.0)	(0.9)	(0.4)	N. f.	(0.7)
*Babesia microti*	1/81	1/67		2/148	1/147	7/135		8/282	1/113	1/39		2/152	3/341	9/241		12/582
	(1.2)	(1.5)	N. f.	(1.4)	(0.7)	(5.2)	N. f.	(2.8)	(0.9)	(2.6)	N. f.	(1.3)	(0.9)	(3.7)	N. f.	(2.1)
Total	2/81	1/67		3/148	1/147	7/135		8/282	3/113	2/39		5/152	6/341	10/241		16/582
	(2.5)	(1.5)	N. f.	(2.0)	(0.7)	(5.2)	N. f.	(2.8)	(2.7)	(5.1)	N. f.	(3.3)	(1.8)	(4.1)	N. f.	(2.7)

*F* females, *M* males, *N* nymphs, *T* total, *N. f.* not found

A total of 54 positive samples were sequenced (one sample was co-infected). Twenty-four samples from *I. ricinus* ticks showed a high level of similarity to *B. microti* (accession numbers: KM051833.1, KM051836.1). *B. venatorum* was confirmed in ten samples (accession numbers: JQ929917.1, KM244044.1). Two samples (accession numbers: KC465977.2, AY572456.1) were identified as *B. divergens*. In three cases, the sequencing failed and *Babesia* species remained unidentified.

In most cases, isolates obtained from *D. reticulatus* ticks showed 100 % similarity to *B. microti* (accession numbers: AB085191.1, AB366158.1). Three samples were defined as *B. canis* (accession numbers: AY072926.1, KM111283.1), and in one isolate, two sequences of *B. canis* and *B. microti* (accession numbers: AY072926.1 and AB085191.1) were found.

## Discussion

The presented results demonstrate that 4.6 % of *I. ricinus* ticks collected on the territory of the Lublin region (eastern Poland) are infected with *Babesia* hemoprotozoans, which confirms that in Poland, there is a potential risk of babesiosis from exposure to the bite of this very common tick species, a competent vector of the disease. Values similar to the presented study, ranging from 3.5 to 4.1 % were recorded in ticks of this species in Germany by Eshoo et al. (2014) and Silaghi et al. (2012), respectively. Studies in other European countries revealed a lower prevalence of *Babesia* in *I. ricinus* compared to the current study, with values ranging from 0.3 % in Hungary (Egyed et al. 2012) to 2.7 % in Belgium (Lempereur et al. 2011). Higher values ranging from 6.1 to 51.7 % were reported from France (Halos et al. 2005; Cotté et al. 2010), Germany (Franke et al. 2011), the Netherlands (Tijsse-Klasen et al. 2011), and Austria (Blaschitz et al. 2008).

*B. microti* distinctly prevailed among *Babesia* species detected in the current study in *I. ricinus* ticks, amounting to 61.5 % of the total count. A similar or higher prevalence of this species in *I. ricinus* was reported from Slovenia (Duh et al. 2001), from Germany (Silaghi et al. 2012; Eshoo et al. 2014), and from Belarus (Reye et al. 2013). All but one *Babesia* species were identified as *B. microti* in the Netherland (96.4 % of all positive *Babesia* spp. ticks) (Tijsse-Klasen et al. 2011).

Different results were obtained by many other authors, mostly from Western and Northern Europe and, less frequently, from central and eastern Europe, who reported the dominancy of *B. venatorum* among *Babesia* species determined in *I. ricinus* ticks. The dominance of *B. divergens* in *I. ricinus* ticks was confirmed by Overzier et al. (2013), Otranto et al. (2014).

It is evident from the above presented results that with only a few exceptions, the *I. ricinus* ticks living in eastern Europe, including Poland, harbor mostly *B. microti*, while those living in western and northern Europe harbor mostly *B. venatorum*. Germany is a transitory area where ticks of this species harbor, usually in almost equal parts, *B. microti* and *B. venatorum* and/or *B. divergens*. This regularity could probably be explained by the fact that in the countries of eastern Europe, the prevalence of *B. microti* in rodents is 10–20 %, which is distinctly higher compared to the countries of western Europe. In consequence, higher infection rates of ticks with *B. microti* could be determined in eastern Europe (Siński et al. 2006; Hartelt et al. 2008). The common occurrence of *B. microti* in *I. ricinus* ticks living on the territory of Poland and other countries of eastern Europe has also been shown by a number of earlier studies where only *B. microti* was determined (Rudolf et al. 2005; Wójcik-Fatla et al. 2006).

The prevalence of *Babesia* spp. in *D. reticulatus* ticks noted in this study was 2.7 %, being lower compared to that found in *I. ricinus*. Similar to *I. ricinus*, also in *D. reticulatus*, the *B. microti* strains prevailed. In Poland, the presence of *B. microti* in adult *D. reticulatus* ticks collected from vegetation has been detected so far only by Wójcik-Fatla et al. (2012) with frequency of 4.5 %. The present study is the first confirmation of these findings, with the slightly lower incidence. To date, the authors of other studies performed in Belgium (Cochez et al. 2012), Germany (Silaghi et al. 2012; Najm et al. 2014), France (Bonnet et al. 2013), Belarus (Reye et al. 2013), and Slovakia (Švehlová et al. 2014) have detected neither the presence of *B. microti* nor other *Babesia* species pathogenic for humans in adult *D. reticulatus* ticks.

The repeatedly found occurrence of *B. microti* in the adult *D. reticulatus* ticks stated in the current study suggests that this species should be considered as a potential vector of human babesiosis, although its role needs an experimental confirmation (Hildebrandt et al. 2013). So far, Walter (1982) has not been successful in the experimental transmission of *B. microti* into golden hamsters by infected *D. reticulatus* nymphs. However, to solve unequivocally the problem of potential risk, such an experiment should be repeated with the adult ticks which are known to feed on humans and large animals. A small percent of the *D. reticulatus* ticks (0.7 %) examined in the presented study harbored *B. canis*, an important causative agent of babesiosis in dogs. *D. reticulatus* is a known vector of this pathogen, and its presence in ticks from eastern Poland is in accordance with the results of Adaszek et al. (2011) that canine babesiosis occurs more often in eastern Poland than in other parts of the country.

Genus *Babesia* spp., as a tick-borne protozoan parasite developing in erythrocytes, could led to rare but potentially life-threatening parasitic disease, which is confirmed by reported clinical cases of babesiosis. The first two cases of this disease in Poland was described by Welc-Falęciak et al. (2010) as a co-infection with Lyme borreliosis, caused by a parasite with a homology of 98.9 % to *B. divergens* or *B. venatorum*. In Europe, before the aforementioned study, only three clinical cases caused by *B. microti* were described, which is a minority of the circa. Fifty cases of human babesiosis recorded in continental Europe, caused mostly by *B. divergens* and, to a less extent, by *B. venatorum* in immunocompromised individuals (Hildebrandt and Hunfeld 2014). The number of babesiosis cases caused by *B. microti* in Europe forms only a small fraction of those reported from North America (Yabsley and Shock 2012). The reason for this discrepancy remains unclear.

## Conclusion

In conclusion, the current study reveals that the population of *I. ricinus*, a competent vector of human babesiosis occurring on the territory of eastern Poland, is infected with a relatively marked frequency with three species of *Babesia* pathogenic for humans, which creates the risk of babesiosis in persons exposed to tick bite. The population of *D. reticulatus*, another tick species inhabiting this territory, is also infected with *B. microti*, and its potential role in spreading the disease should be considered and further investigated by experimental studies.
